# Supplementary material for: Cleavage of α-1,4-glycosidic linkages by the glycosylphosphatidylinositol-anchored α-amylase AgtA decreases the molecular weight of cell wall α-1,3-glucan in Aspergillus oryzae
Source: Front Fungal Biol. 2023 Jan 10;3:1061841. doi: 10.3389/ffunb.2022.1061841 (PMC10512346; doi:10.3389/ffunb.2022.1061841)
Supplement: Supplementary file 1 [file DataSheet_1.pdf]

Supplementary Material

MATERIALS AND METHODS

Enzymatic Synthesis of *p*-Nitrophenyl α-Maltooligosides

A mixture (530 μL) containing Mal_5-α-*p*NP (100 mg, 200 mM) and 180 mU/mL rAoAgtA in dialyzed culture supernatant in 50 mM Na-Ac buffer (pH 5.5) was incubated at 40°C for 24 h. The reaction was stopped by adding 11 mL of methanol. The reaction mixture was evaporated, dissolved in a small amount of H_2O, and then applied to a Toyopearl HW-40S column (4.0 × 60 cm) equilibrated with H_2O at a flow rate of 0.6 mL/min. The eluate was collected in 10-mL fractions (950 mL in total). Each fraction was analyzed by HPLC. The HPLC system consisted of a Mightysil Si60 column (4.6 × 250 mm; Kanto Chemical, Tokyo, Japan) and a Jasco Intelligent System Liquid Chromatograph; detection was performed at 300 nm. The bound material was eluted with 75% acetonitrile at a flow rate of 1.0 mL/min at 40°C. Fractions corresponding to $Mal_{2–8}$-α-*p*NP were concentrated and then lyophilized. Products with a purity of less than 98% were rechromatographed under the same conditions. As a result, Mal_8-α-*p*NP (4.7 mg, yield 3.1%), Mal_7-α-*p*NP (5.5 mg, 4.1%), Mal_6-α-*p*NP (7.1 mg, 6.1%), Mal_5-α-*p*NP (12.3 mg, 12.3%), Mal_4-α-*p*NP (6.2 mg, 7.5%), Mal_3-α-*p*NP (10.8 mg, 16.4%), and Mal_2-α-*p*NP (11.9 mg, 24.4%) were obtained. The structures of the synthesized $Mal_{2–8}$-α-*p*NP were evaluated by ^{1}H NMR analysis in D_2O (Supplementary Figure 3); 500 MHz ^{1}H NMR spectra were recorded using a Jeol ECX-500 II spectrometer (Jeol, Akishima, Japan).

Biochemical Characterization of Recombinant AoAgtA

Purified rAoAgtA was used in all tests except that for pH stability, where dialyzed culture supernatant was used.

The optimum temperature was determined within a range of 4–60°C in 50 mM Na-Ac buffer (pH 5.5) (Supplementary Figure 6A). To measure thermostability, the enzyme solutions were heated at 10–70°C for 30 min and then residual rAoAgtA activity was measured (Supplementary Figure 6B).

The optimum pH was determined at 40°C in the following 50 mM buffers: glycine-HCl (pH 1–3), citric acid-NaOH (pH 3–4), Na-Ac (pH 4–6), 3-morpholinopropanesulfonic acid (MOPS)-NaOH (pH 6–7), Tris-HCl (pH 7–9), and glycine-NaOH (pH 9–10) (Supplementary Figure 6C). To measure pH stability, enzyme solutions were incubated at 4°C for 30 min in 10 mM each buffer mentioned above and then residual rAoAgtA activity was measured (Supplementary Figure 6D).

The effect of metal ions (LiCl, KCl, $MgCl_2$, $CaCl_2$, $MnCl_2$, $FeCl_3$, $CoCl_2$, $CuCl_2$, $ZnCl_2$, and $AlCl_3$) and EDTA on rAoAgtA activity was evaluated by measuring rAoAgtA activity in 50 mM Na-Ac buffer (pH 5.5) in the presence of 2.5 mM of each metal ion or EDTA; rAoAgtA activity in their absence was considered 100% (Supplementary Table 4).

We also evaluated the effect of $CaCl_2$ on rAoAgtA activity after treatment with EDTA (Supplementary Table 5). The enzyme (0.19 mU) and 25 mM EDTA (2 μL; 2.5 mM at final concentration) were incubated in 63 mM Na-Ac buffer (pH 5.5; 50 mM at final concentration) at 4°C for 30 min without the substrate. Next, the substrate (Mal_5-α-*p*NP) was added, and rAoAgtA activity

was measured. Alternatively, 25 mM $CaCl_2$ (2 μL; 2.5 mM at final concentration) was added after the incubation with EDTA, and the sample was incubated at 4°C for another 30 min. Mal_5-α-*p*NP was then added, and rAoAgtA activity was measured.

Supplementary Table 1. Primers used in this study.

Purpose	Primer name	Sequence (5′ to 3′)
Construction of *agtA*^{OE} and Δ*agtA* strains of *Aspergillus oryzae*		
	agtA-Fw-NotI	GACAAGCTTGCGGCCGCATGGTTTCGTCGTCATCCCT
	agtA-Rv-NotI	AGTCACGTGGCGGCCGCCTACAACAATACCGCAACAAGAC
	agtA-up-Fw	TCCTTCCAACACCGATCCAG
	agtA-mid-Rv	TGCTGGCGTCGGTACATACC
	agtA-LU	CCTTCTTTTCCCGTCCTT
	agtA-LL+adeA	ATATACCGTGACTTTTTAGTGAAAGTAACTGGAGTCGT
	agtA-RU+adeA	AGTTTCGTCGAGATACTGCCGGAGAAGTTCTTCAGCA
	agtA-RL	TCGGCTAAGTTACAGACAG
	agtA-AU	GACTCCAGTTACTTTCACTAAAAAGTCACGGTATATCATGAC
	agtA-AL	TGCTGAAGAACTTCTCCGGCAGTATCTCGACGAAACTACCTAA
Construction of the rAoAgtA expression system		
	agtA-Fw-NdeI	GGAATTCCATATGGTTTCGTCGTCATCCCT
	agtA-Rv-SmaI	CCCCCGGGCGCCGCGCCCTTGGTCTTCAG
	agtA-Fw-PstI	ATCAGCCGCTGCAGCAACCACAGCAGAATGGAAG
	agtA-Rv-XbaI	CCAGTGTGTCTAGATTCGCCCTTTTATTAATGA
Construction of heterologous *agtA*^{OE} strains of *Aspergillus nidulans*		
	agtA-dIntron-Fw	TATGGCGCTTGCTAAGAATGTTTTAACCTTCAC
	agtA-dIntron-Rv	TTAGCAAGCGCCATATCATCAGTCATACTAGCG
	agtA-IF-top-Fw	CGCACCACCTTCAAAATGGTTTCGTCGTCATCCCTG
	TagdA-IF-tail-Rv	TTGTGCTTCTCTGCAAGGTGTACGCTTGGTAAAGTTG
	AopyrG-IF-Right-Fw	TGCAGAGAAGCACAATTTCCTCATC
	Ptef1-tail-Rv	TTTGAAGGTGGTGCGAACTTTGTAG
	397-5	GAGGCCACTCAGGCCGATATCACC
	ANamyD-up-Fw	AGGTTCAACGATCGAACCCAGCAAC
	Hph-top-Rv	CCAGCTTGTGTTCCCGGTCTG
	AopyrG-IF-Right-Rv	GCCAGTGAATTCGAGCTCAACTGCACCTCAGAAGAAAAGGATG

Supplementary Table 2. [1]H-chemical shifts and coupling constants of the anomeric protons of 3-α-maltosyl-, 3-α-maltotriosyl-, and 3-α-maltotetraosyl-glucose dissolved in D_2O (25°C).

Compound	H1		H1'		H1''		H1'''		H1''''	
	α	β	α	β	α	β	α	β	α	β
	J, Hz	*J*, Hz	*J*, Hz	*J*, Hz	*J*, Hz	*J*, Hz	*J*, Hz	*J*, Hz	*J*, Hz	*J*, Hz
3-α-Maltosylglucose (Koto et al., 1992)	5.15	4.59	5.28	5.30	5.33	5.33				
	3.5	8.0	4.0	4.0	3.5	3.5				
3-α-Maltosylglucose	5.16	4.59	5.29	5.30	5.34	5.34				
	3.8	8.1	3.9	3.9	3.9	3.9				
3-α-Maltotriosylglucose	5.16	4.59	5.29	5.31	5.33	5.33	5.32	5.32		
	3.8	8.0	3.9	3.9	4.0	4.0	4.0	4.0		
3-α-Maltotetraosylglucose	5.16	4.59	5.29	5.31	5.34	5.34	5.32	5.32	5.31	5.31
	3.8	8.0	3.9	3.9	4.0	4.0	3.9	3.9	3.9	3.9

For each compound: upper row, [1]H-chemical shifts; lower row, coupling constants (Hz).

Supplementary Table 3. ^{13}C-chemical shifts of 3-α-maltosyl-, 3-α-maltotriosyl-, and 3-α-maltotetraosyl-glucose dissolved in D_2O (25°C).

Carbon number	Compounds			
	3-α-Maltosylglucose (Koto et al., 1992)	3-α-Maltosylglucose	3-α-Maltotriosylglucose	3-α-Maltotetraosylglucose
1α	93.2	93.2	93.2	93.2
1β	96.9	96.9	96.9	96.9
2α	71.1	71.1	71.1	71.1
2β	73.8	73.8	73.8	73.8
3α	80.5	80.5	80.4	80.4
3β	83.1	83.1	83.1	83.1
4	71.0	71.0	71.0	71.0
5α	72.2	72.2	72.2	72.2
5β	76.6	76.6	76.6	76.6
6α	61.2	61.2	61.1	61.1
6β	61.6	61.5	61.5	61.5
1'	99.8	99.8	99.8	99.8
2'α	72.5	72.5	72.5	72.5
2'β	72.4	72.4	72.4	72.4
3'	74.3	74.3	74.3	74.3
4'α	77.7	77.7	77.8	77.7
4'β	77.6	77.6	77.7	77.6
5'	71.3	71.3	71.2	71.2
6'	61.4	61.3	61.3	61.3
1''	100.6	100.6	100.4	100.4
2''α	72.7	72.7	72.5	72.5
2''β			72.1	72.1
3''	73.8	73.9	74.3	74.3
4''	70.3	70.3	77.7	77.6
5''	73.7	73.6	71.0	71.0
6''	61.5	61.4	61.4	61.4
1'''			100.7	100.6
2'''α			72.7	72.5
2'''β				72.2
3'''			73.8	74.3
4'''			70.3	77.9
5'''			73.7	71.0
6'''			61.4	61.4
1''''				100.7
2''''				72.7
3''''				73.8
4''''				70.3
5''''				73.7
6''''				61.4

Supplementary Table 4. Effect of metal ions and EDTA on rAoAgtA activity.

Chemical	Relative activity (%)
Control	100 ± 6
Li^{+}	105 ± 3
K^{+}	102 ± 1
Mg^{2+}	100 ± 3
Ca^{2+}	111 ± 3
Mn^{2+}	110 ± 12
Fe^{3+}	63 ± 3
Co^{2+}	92 ± 11
Cu^{2+}	n.d.
Zn^{2+}	56 ± 6
Al^{3+}	101 ± 4
EDTA	21 ± 6

rAoAgtA activity in the absence of metal ions and EDTA was considered as 100%.

n.d., not detected (no activity).

Data are mean ± standard deviation of three replicates.

Supplementary Table 5. rAoAgtA activity in the presence of EDTA and Ca^{2+}.

Chemicals	Relative activity (%)
Control	100 ± 6
+EDTA	13 ± 1
+EDTA, Ca^{2+}	93 ± 5

rAoAgtA activity in the absence of EDTA and Ca^{2+} was considered as 100%.

Data are mean ± standard deviation of three replicates.

Supplementary Table 6. Degradation of various natural glucans by rAoAgtA.

Substrate	Glycosidic linkage	Degradation activity
Corn starch	α-1,4 (primary), α-1,6	-
Potato starch	α-1,4 (primary), α-1,6	-
Soluble starch	α-1,4 (primary), α-1,6	+, 3.23 ± 0.28 mU/mL
Dextran	α-1,4, α-1,6 (primary)	-
Pullulan	α-1,4, α-1,6	-
α-1,3-Glucan (bacterial)	α-1,3	-
Nigeran	α-1,3, α-1,4	-
Cellulose	β-1,4	-
Pustulan	β-1,6	-
Laminaran	β-1,3 (primary), β-1,6	-

+, degraded; -, not degraded

Data are mean ± standard deviation of three replicates.

Supplementary Table 7. Four highly conserved regions of enzymes belonging to the α-amylase family.

Enzyme	Origin	Sequence in				NCBI accession number
		Region I	Region II	Region III	Region IV	
Agt protein						
AoAgtA	*Aspergillus oryzae*	140 DTVINN	229 GLRIDAAKH	257 EVLQ	318 FSENHD	XP_001820542
AnAgtA	*Aspergillus niger*	142 DTVINN	231 GLRIDAAKH	259 EVLQ	320 FSENHD	XP_003188777
AmyD	*Aspergillus nidulans*	141 DTVINN	230 GLRIDAAKH	258 EVLQ	319 FSENHD	XP_660912
α-Amylase (Taka-amylase A)	*Aspergillus oryzae*	138 DVVANH	223 GLRIDTVKH	251 EVLD	313 FVENHD	P0C1B3
CGTase	*Paenibacillus macerans*	162 DFAPNH	252 GIRFDAVKH	285 EWFL	351 FIDNHD	P31835
Pullulanase	*Klebsiella aerogenes*	619 DVVYNH	690 GFRFDLMGY	723 EGWD	846 YVSKHD	P07811
Isoamylase	*Pseudomonas amyloderamosa*	318 DVVYNH	397 GFRFDLASV	461 EPWA	531 FIDVHD	P10342
Branching enzyme	*Escherichia coli*	335 DWVPGH	401 ALRVDAVAS	458 EEST	521 LPLSHD	P07762
Neopullulanase	*Geobacillus stearothermophilus*	242 DAVFNH	324 GWRLDVANE	357 EIWH	419 LLGSHD	P38940
Amylopullulanase	*Thermoanaerobacter pseudethanolicus*	519 DGVFNH	624 GWRLDVANE	657 ELWG	729 LLGSHD	P38939
α-Glucosidase	*Saccharomyces cerevisiae*	106 DLVINH	210 GFRIDTAGL	276 EVAH	344 YIENHD	P07265
Cyclomaltodextrinase	*Thermoanaerobacter thermohydrosulfuricus*	238 DAVFNH	321 GWRLDVANE	354 EVWH	416 LIGSHD	A42950
Oligo-1,6-glucosidase	*Bacillus cereus*	98 DLVVNH	195 GFRMDVINF	255 EMPG	324 YWNNHD	P21332
Dextran glucosidase	*Streptococcus mutans*	93 DLVVNH	185 GFRMDVIDM	231 ETWG	303 FWNNHD	Q2HWU5

The three catalytic residues are shaded in gray. The His residue in Region I conserved among the members of the α-amylase family but replaced with Asn in Agt proteins are highlighted in blue. Numbering of the amino acid sequences of the enzymes starts at N-terminal amino acid of each enzyme including signal peptide.

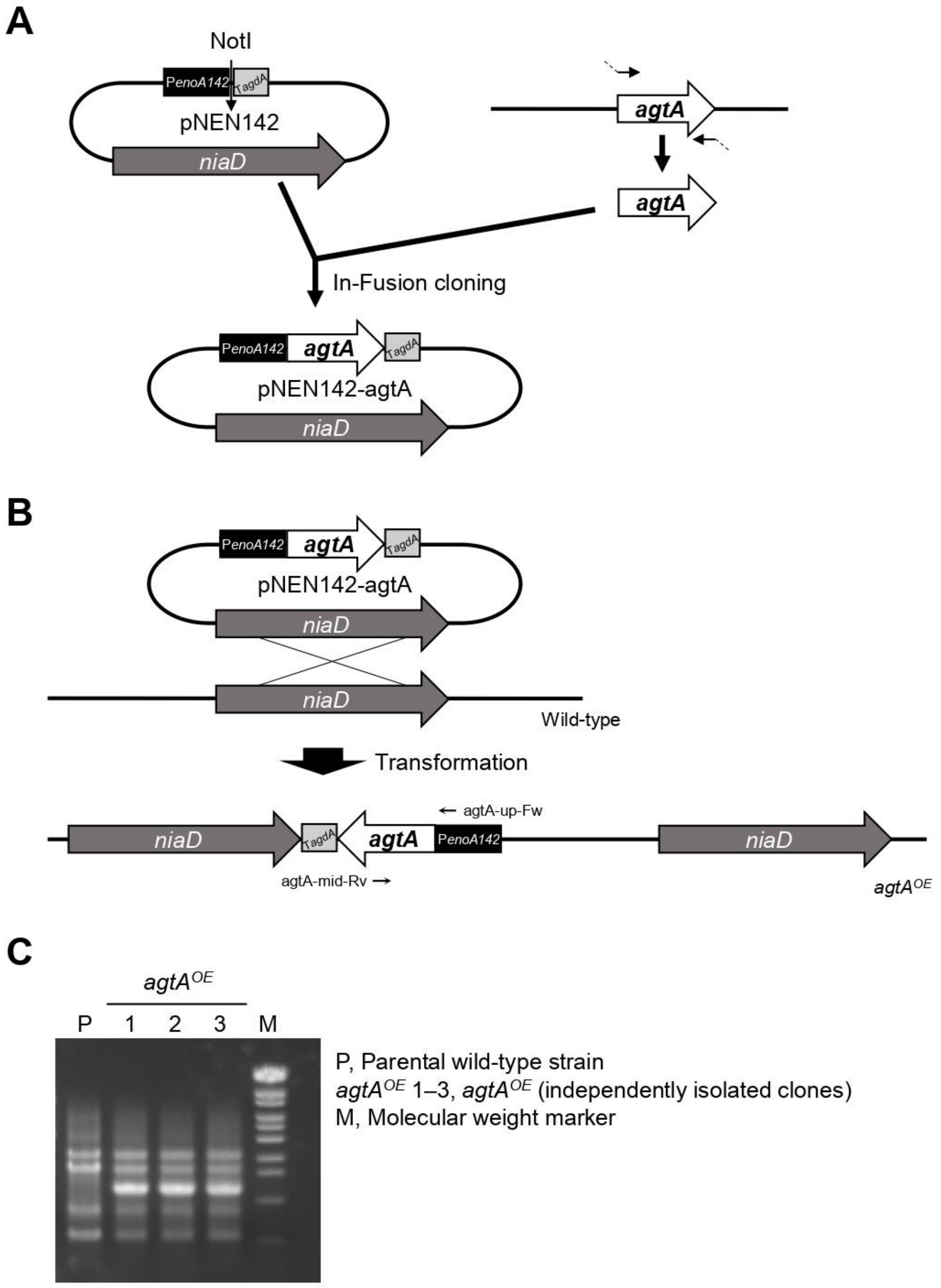

Supplementary Figure 1. Construction of *agtA*^{OE} strain of *A. oryzae*. (A) Construction of the pNEN142-agtA plasmid. **(B)** Strategy for *agtA* overexpression. pNEN142-agtA was used to transform the wild-type strain. **(C)** PCR analysis with primers shown in **B** confirmed the integration of the *agtA* overexpression cassette.

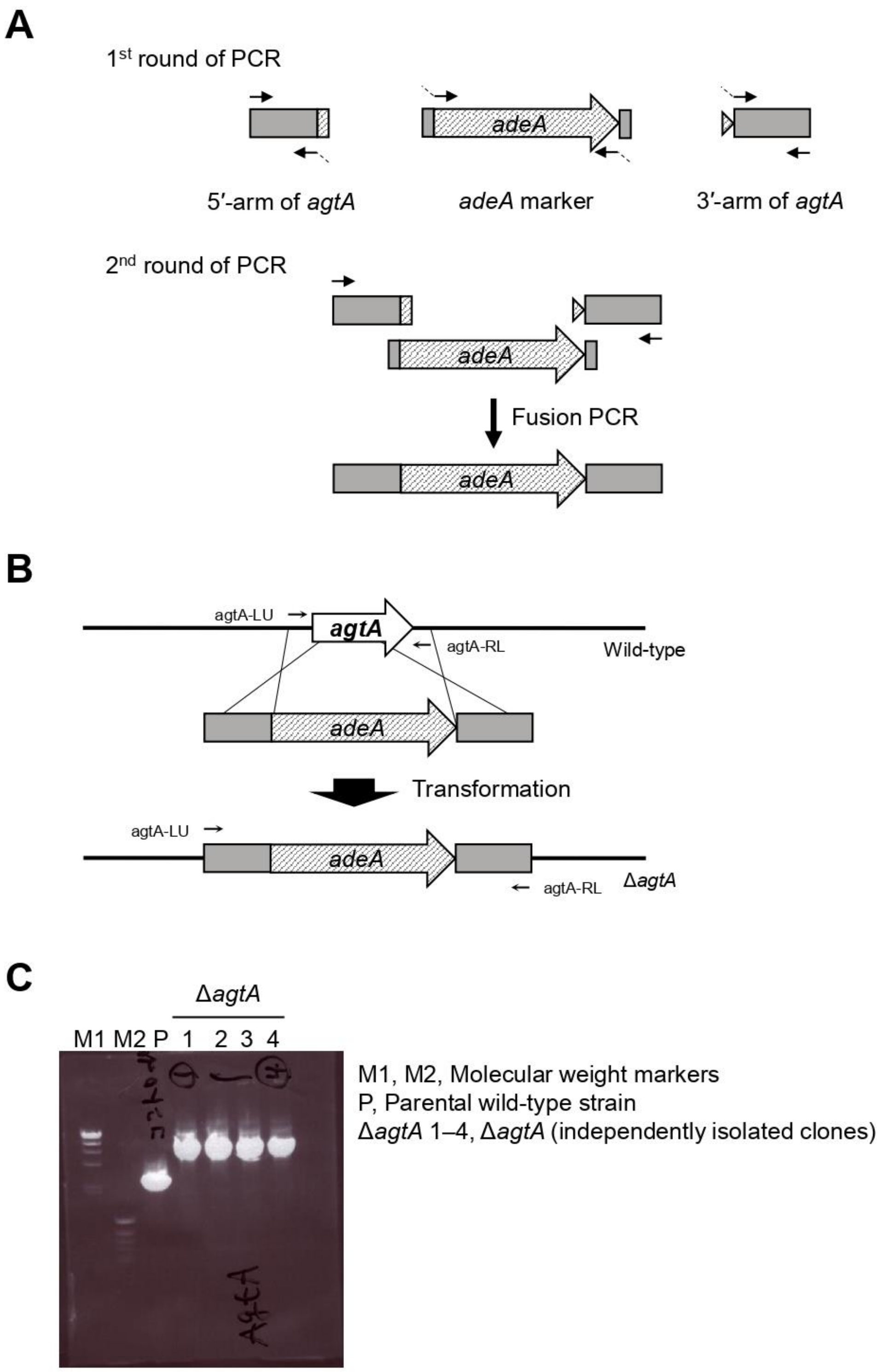

Supplementary Figure 2. Construction of Δ*agtA* strain of *A. oryzae*. (A) Construction of the *agtA* disruption cassette. Fragments containing the 5′- and 3′-arms of *agtA* for gene replacement and the *adeA* marker were amplified, and then the three fragments were fused. **(B)** Strategy for *agtA* disruption. The fused cassette was used to transform the wild-type strain. **(C)** PCR analysis of *agtA* gene disruption with primers shown in **B**.

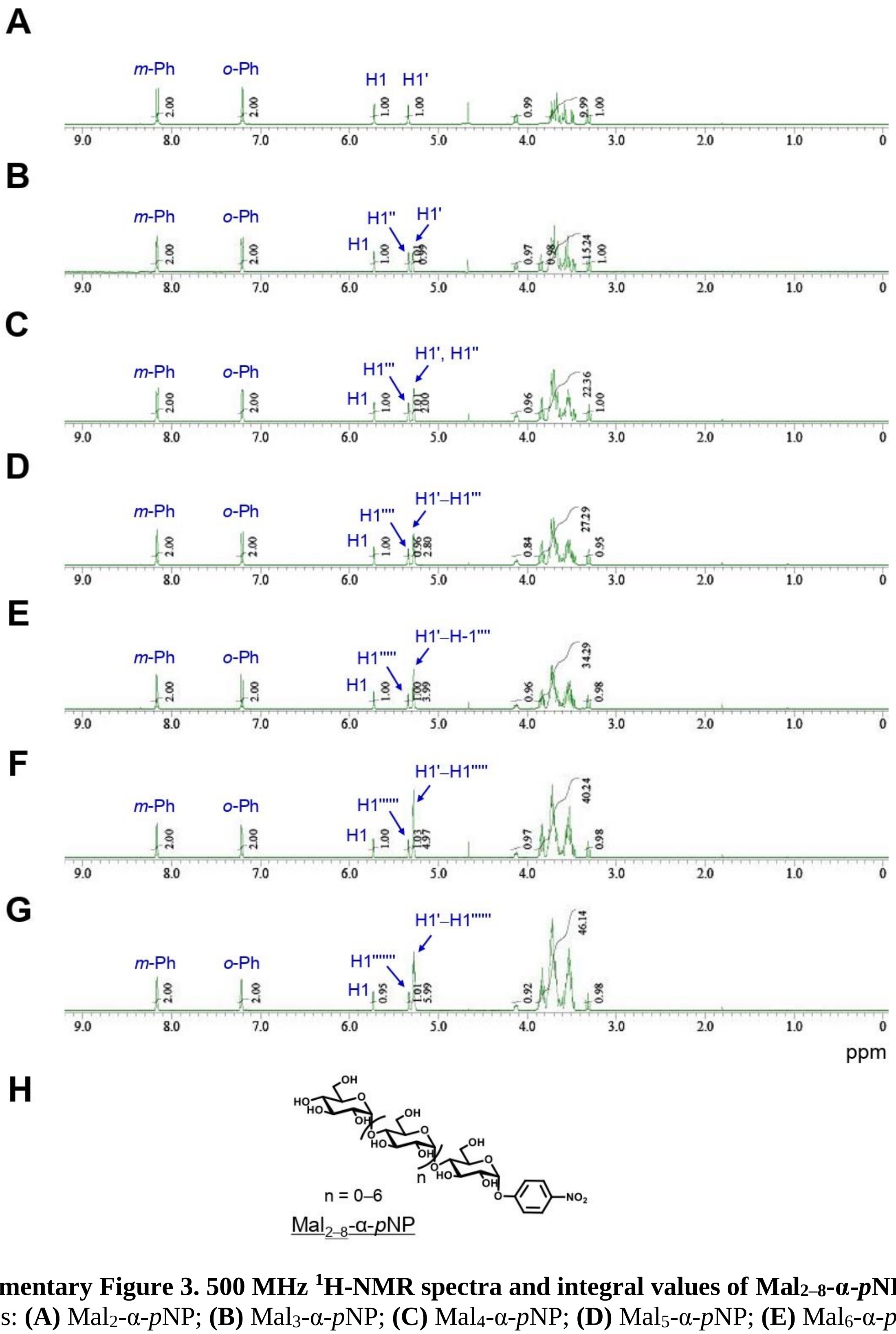

Supplementary Figure 3. 500 MHz ^{1}H-NMR spectra and integral values of Mal$_{2–8}$-α-*p*NP. Samples: **(A)** Mal$_2$-α-*p*NP; **(B)** Mal$_3$-α-*p*NP; **(C)** Mal$_4$-α-*p*NP; **(D)** Mal$_5$-α-*p*NP; **(E)** Mal$_6$-α-*p*NP; **(F)** Mal$_7$-α-*p*NP; **(G)** Mal$_8$-α-*p*NP. **(H)** Chemical structures of Mal$_{2–8}$-α-*p*NP. Solvent, D_2O; temperature, 25°C; concentration, 5 mg/620 μL.

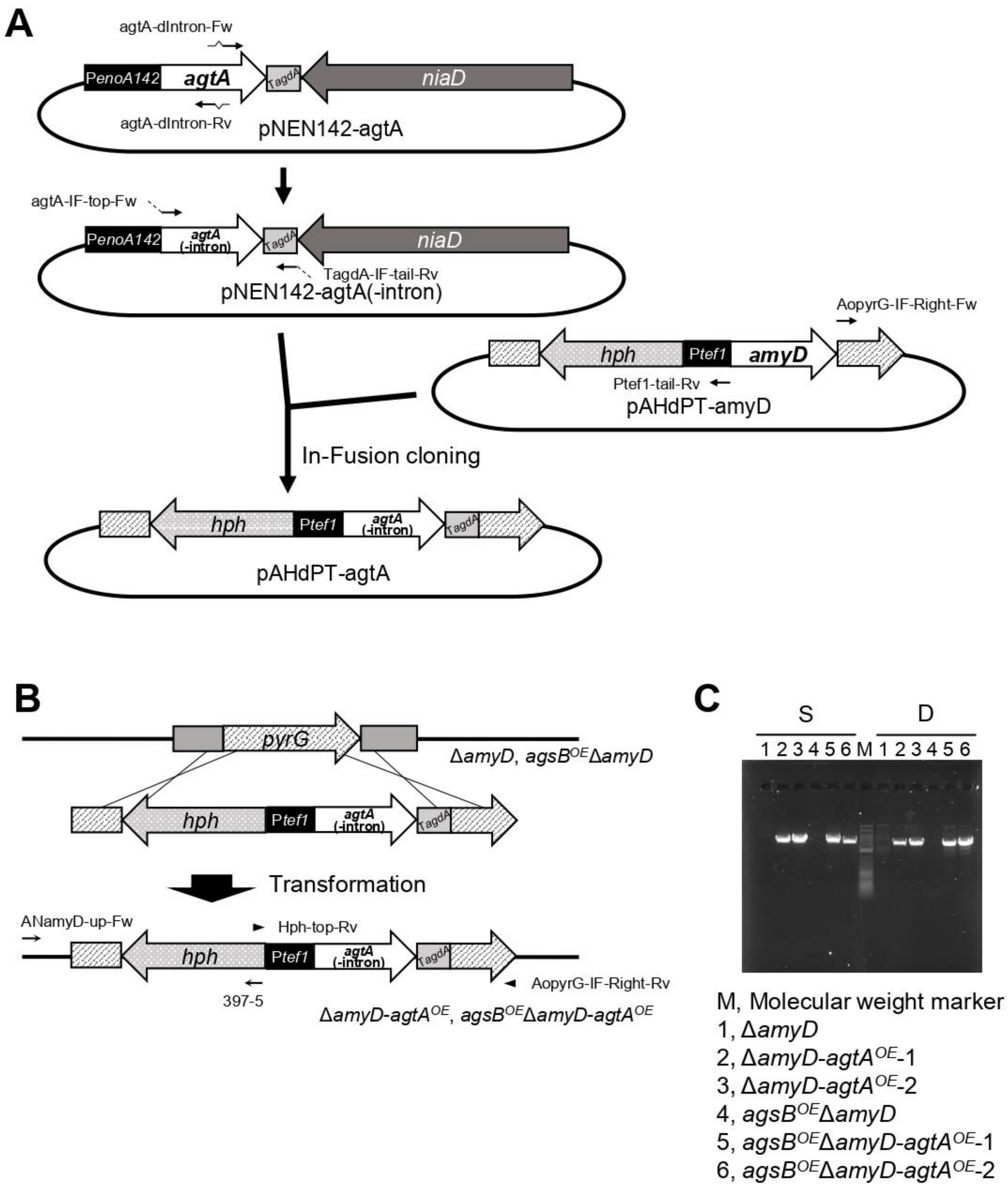

Supplementary Figure 4. Construction of *agtA*^{OE} strains of *A. nidulans*. (A) Construction of the pAHdPT-agtA plasmid. The intron of *agtA* was removed from pNEN142-agtA to obtain pNEN142-agtA(-intron). PCR amplification was performed with pNEN142-agtA(-intron) and pAHdPT-amyD as templates, and the two fragments were fused. **(B)** Strategy for overexpression of *agtA* in *A. nidulans*. SacI-digested pAHdPT-agtA was used to transform the Δ*amyD* and *agsB*^{OE}Δ*amyD* strains. **(C)** PCR analysis with two pairs of primers shown in **B** (S, arrows; D, arrowheads) confirmed the integration of the *agtA* overexpression cassette.

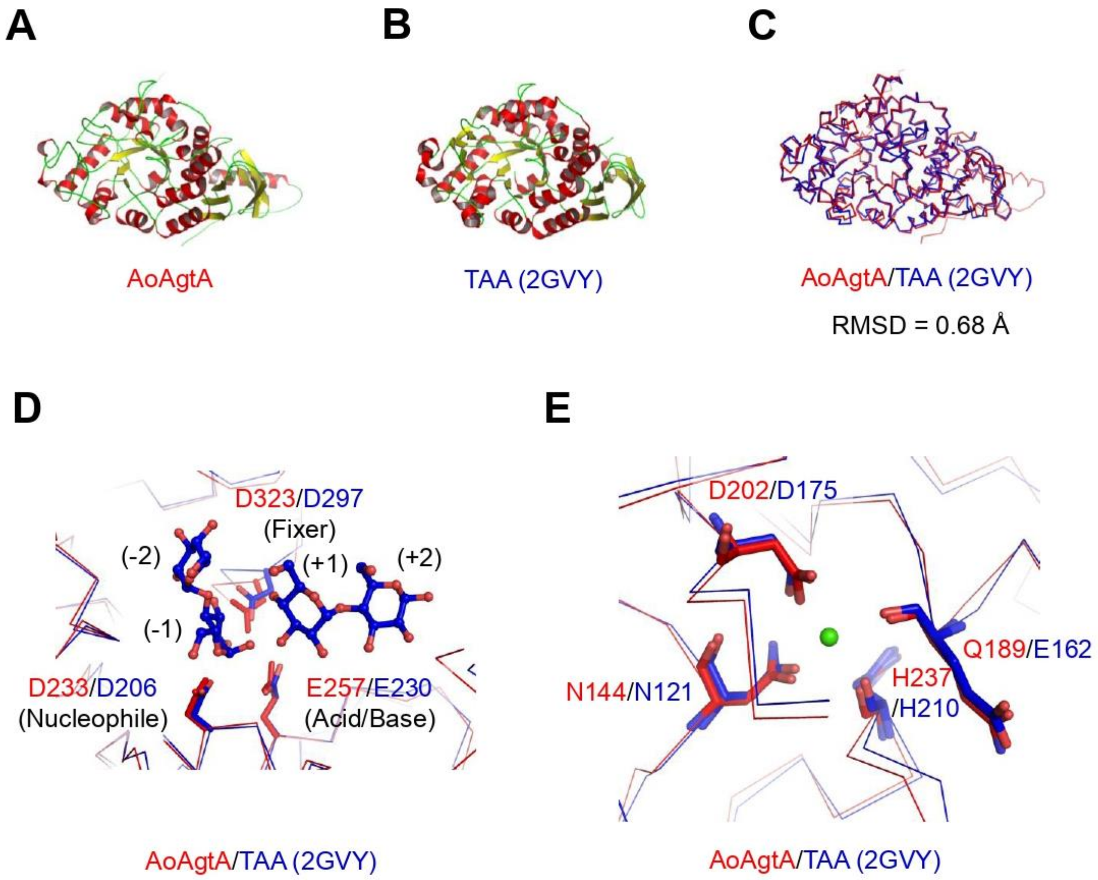

Supplementary Figure 5. Structure of AoAgtA predicted by AlphaFold2. (A) The predicted structure of AoAgtA. **(B)** Crystal structure of Taka-amylase A (TAA) (PDB, 2GVY). **(C)** AoAgtA superimposed on TAA. RMSD, root mean square distance between the Cα atoms of 2 aligned residues. **(D)** Catalytic residues and **(E)** Ca^{2+}-binding residues of AoAgtA and TAA are conserved. Bound maltose and Ca^{2+} in TAA are shown as ball-and-stick models. Numbers in parentheses indicate the subsites in TAA (**D**). In the panels **D** and **E**, numbering of the amino acid residue starts at the N-terminal of AoAgtA including its signal peptide, and dose that at the N-terminal of TAA without signal peptide.

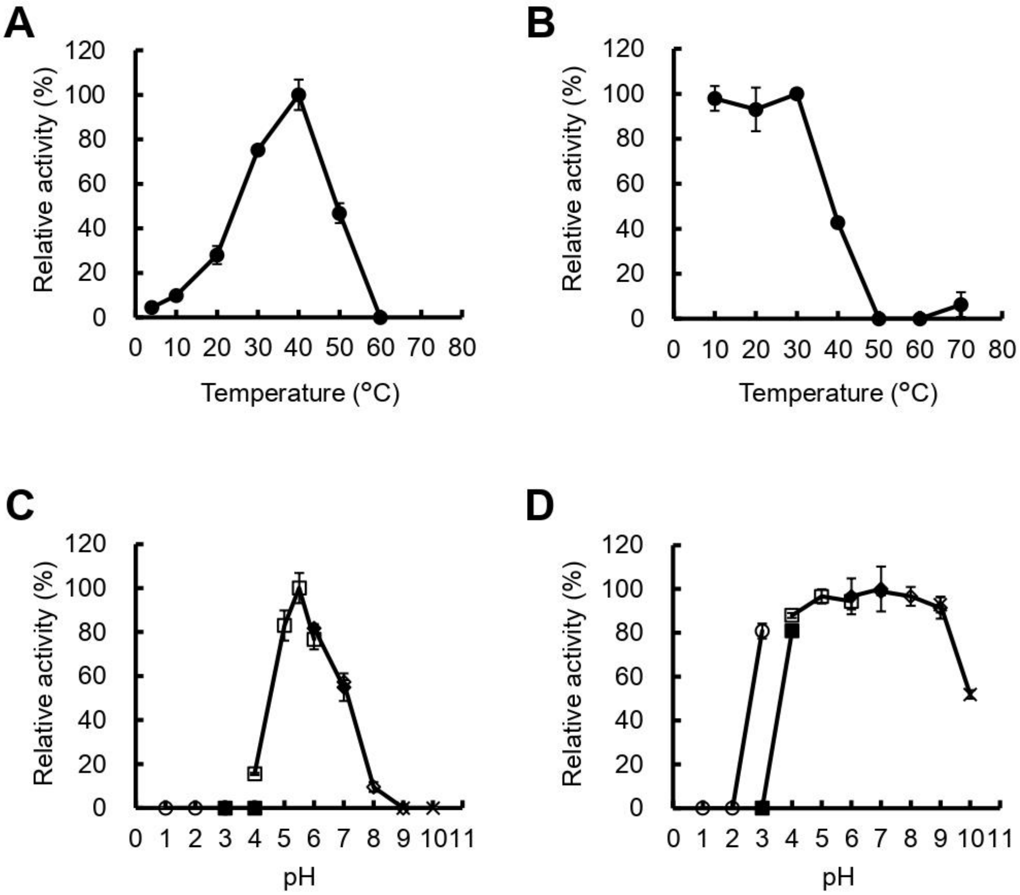

Supplementary Figure 6. Biochemical characterization of rAoAgtA. **(A)** Optimal temperature. **(B)** Thermostability. **(C)** Optimal pH. **(D)** pH stability. A mixture (20 μL) containing 1 mM Mal_5-α-*p*NP and appropriate rAoAgtA in (**A, B**) 50 mM sodium acetate (Na-Ac) buffer (pH 5.5) or (**C, D**) the indicated buffer and pH was incubated at (**C, D**) 40°C or (**A, B**) the indicated temperature for 10 min. Samples were analyzed by HPLC, and the amount of Mal_2-α-*p*NP was quantified to calculate the rAoAgtA activity. Each graph shows relative enzymatic activity, with the highest activity considered 100%. Effect of pH on rAoAgtA activity and stability was examined in different buffers: glycine-HCl (○), citric acid-NaOH (■), Na-Ac (□), MOPS-NaOH (♦), Tris-HCl (◊), and glycine-NaOH (×). Error bars represent the standard deviation of the mean calculated from three replicates.

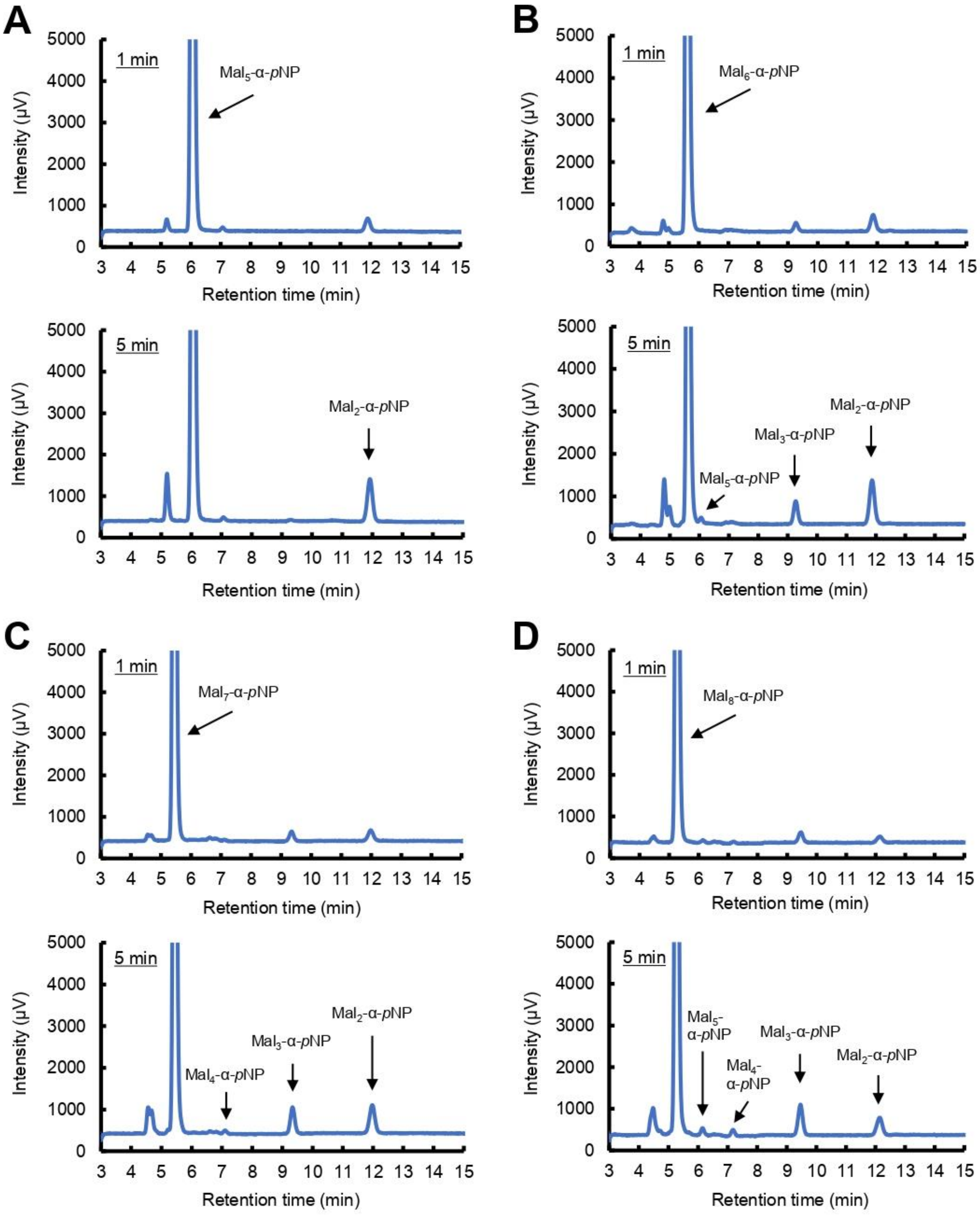

Supplementary Figure 7. HPLC chromatograms of the substrates and products of Mal$_{5–8}$-α-*p*NP degradation by rAoAgtA. Substrates: **(A)** Mal$_5$-α-*p*NP; **(B)** Mal$_6$-α-*p*NP; **(C)** Mal$_7$-α-*p*NP; **(D)** Mal$_8$-α-*p*NP. A mixture (20 µL) containing 1.6 mM each substrate and 9.5 mU/mL rAoAgtA in 50 mM Na-Ac buffer (pH 5.5) was incubated at 40°C for 1 or 5 min.

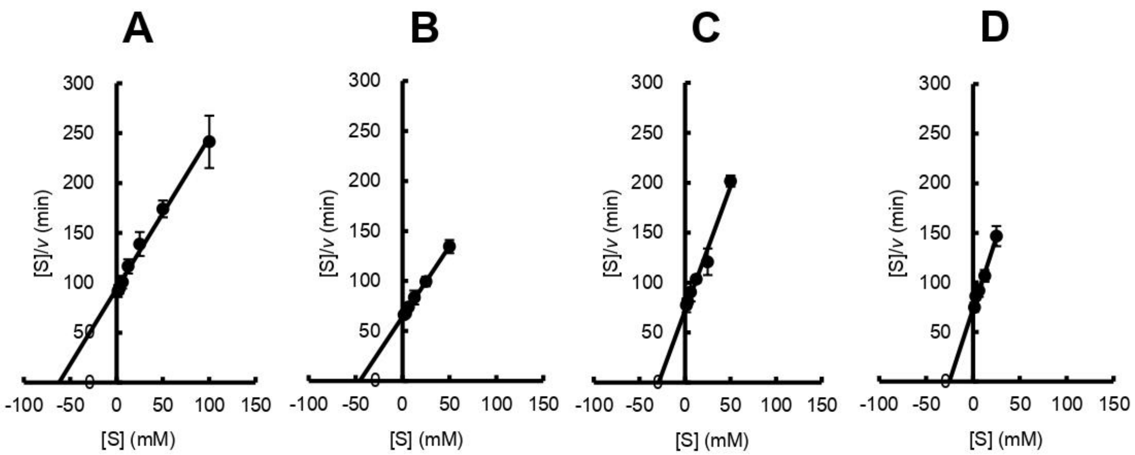

Supplementary Figure 8. Hanes–Woolf plots for Mal$_{5–8}$-α-*p*NP degradation by rAoAgtA. Substrates: **(A)** Mal$_5$-α-*p*NP; **(B)** Mal$_6$-α-*p*NP; **(C)** Mal$_7$-α-*p*NP; **(D)** Mal$_8$-α-*p*NP. The kinetic parameters of the reaction were determined by measuring the initial velocity (v) (5 min) at each substrate concentration ([S]). A linear transform was used to calculate the Michaelis constant (K_m) and maximum velocity (V_{max}) of rAoAgtA. Error bars represent the standard deviation of the mean calculated from three replicates.

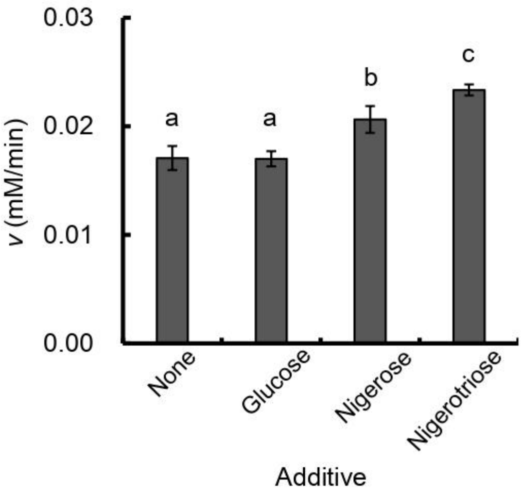

Supplementary Figure 9. **The release velocity of Mal$_2$-*α*-*p*NP from Mal$_5$-*α*-*p*NP by rAoAgtA with or without nigerooligosaccharides.** A mixture (20 μL) containing 1.6 mM Mal$_5$-α-*p*NP, 9.5 mU/mL rAoAgtA, and 16 mM nigerooligosaccharide or glucose in 50 mM Na-Ac buffer (pH 5.5) was incubated at 40°C for 5 min. Samples were analyzed by HPLC, and the amount of Mal$_2$-α-*p*NP was quantified to calculate its release velocity (*v*). Error bars represent the standard deviation of the mean calculated from three replicates. Different letters above bars indicate significant difference by Tukey's test ($P < 0.01$ none or glucose vs nigerose, none or glucose vs nigerotriose; $P < 0.05$ nigerose vs nigerotriose).

REFERENCE

Koto, S., Morishima, N., Shichi, S., Haigoh, H., Hirooka, M., Okamoto, M., et al. (1992). Dehydrative glycosylation using heptabenzyl derivatives of glucobioses and lactose. *Bull. Chem. Soc. Jpn.* 65, 3257–3274. doi: 10.1246/bcsj.65.3257.
